# Expression of the *Nicotiana benthamiana* Retrozyme 1 (NbRZ1) Genomic Locus

**DOI:** 10.3390/plants14081205

**Published:** 2025-04-14

**Authors:** Alexander A. Lezzhov, Anastasia K. Atabekova, Denis A. Chergintsev, Andrey G. Solovyev, Sergey Y. Morozov

**Affiliations:** A. N. Belozersky Institute of Physico-Chemical Biology, Moscow State University, 119992 Moscow, Russia; lezzhov-genetic@mail.ru (A.A.L.); asya_atabekova@mail.ru (A.K.A.); ledumpalustre86@gmail.com (D.A.C.); solovyev@belozersky.msu.ru (A.G.S.)

**Keywords:** retrozymes, retroelements, long terminal repeat, LTR promoter, transcription, ribozymes, circRNA, circRNA translation

## Abstract

Retrozymes are a class of non-autonomous plant retrotransposons that have long terminal repeats (LTRs) containing hammerhead ribozymes (HHRs) that facilitate the circularization of the retrozyme RNA. The LTR of *Nicotiana benthamiana* retrozyme 1 (NbRZ1) has been shown to contain a promoter that directs transcription of this retroelement. In this study, we identified the transcription start site of the promoter contained in the LTR of NbRZ1 and mapped the promoter region essential for its transcriptional activity. Using transgenic *Arabidopsis thaliana* plants carrying the GUS gene under the control of the NbRZ1 LTR, the NbRZ1 transcript was demonstrated to potentially encode a protein targeted for proteasomal degradation in the plant cell. Overexpression of this protein in plants using a viral expression vector was found to cause severe necrosis. The data presented suggest a tight regulation of the expression of the NbRZ1-encoded polypeptide in plants and its potential functional importance, although further research is needed to determine whether circular and/or linear retrozyme RNA forms can be translated in plants.

## 1. Introduction

The functions of circular RNAs (circRNAs) in plants and animals have been the subject of intense research in recent years [1,2]. Most of the circRNAs discovered so far are the result of backsplicing, which was previously thought to be the result of misregulation [3]. However, subsequent research has led to a revision of this concept. Current data indicate that circRNAs have important regulatory functions in cells and that the process of their generation is directed and highly regulated [1]. Different mechanisms can exert different functions of circRNAs. It has been shown that circRNAs are able to regulate transcriptional elongation efficiency by forming RNA-DNA hybrids, called R-loops, with corresponding genomic loci, as demonstrated on the model of a circRNA encoded by exon 6 of the *Arabidopsis thaliana SEPALLATA3* gene [4]. In addition, certain circRNAs have been shown to contain microRNA (miRNA) binding sites and thus act as microRNA sponges [5]. miRNA-sponge function has been studied using the model of ciRS-7 (circular RNA sponge for miR-7) [6], which is expressed in the human brain and, due to the presence of multiple miR-7 binding sites, is able to bind the corresponding miR involved in neurogenesis [7]. Furthermore, it has been demonstrated that translation on the circRNA templates can occur in different systems, although the mechanisms and functional significance of the translated products are not yet fully understood [8,9,10,11]. Nevertheless, it has been shown that the protein synthesized on the template of *Drosophila melanogaster* circRNA called CircSfl is associated with increased lifespan longevity [12].

Another recently discovered type of circRNA is formed by the activity of hammerhead ribozymes (HHRs) followed by ligation [13]. This type of circRNA is represented by the transcripts of retrozymes (retrotransposons with hammerhead ribozymes) found in the genomes of some plant and metazoan species [13,14]. These retroelements exhibit a functional duality. On the one hand, they are non-autonomous retrotransposons integrated into the genome, and on the other hand, the transcripts of these retroelements are circRNA. Plant and animal retrozymes have structural differences. In particular, animal retrozymes are non-autonomous, non-LTR retrotransposons with type I HHRs, whereas plant retrozymes contain type III HHRs and are LTR-containing retrotransposons. For mobilization, retrozymes apparently require appropriate autonomous retroelements encoding proteins necessary for reverse transcription (RT) and integration into a new genomic locus. In the case of plant retrozymes, this may be an autonomous Ty3-gypsy family retroelement, whereas in the case of metazoan retrozymes, this role may be played by non-LTR retrotransposons such as LINEs or Penelope-like retroelements [13,14].

Plant retrozyme genomic loci consist of two long direct terminal repeats (LTRs) of approximately 300–400 nt containing type III HHRs and flanking a central region with a primer binding site complementary to the tRNA-Met 3′-terminal sequence and a polypurine tract, both of which are required for DNA synthesis during the mobilization of LTR retrotransposons [13]. It has been demonstrated that retrozymes replicate by a rolling circle mechanism and that both circular and linear replicative intermediates accumulate in different plant organs [13,15]. However, it remains unclear whether this is the result of active transcription of the corresponding genomic locus or the result of RNA-RNA replication since the LTR sequence containing the promoter is highly methylated, making active transcription of this genomic locus unlikely [15]. In addition, retrozyme RNA has been shown to be capable of long-distance transport in plants, making it similar to viroids, which are circular non-coding RNAs capable of rolling-circle replication and systemic transport in plants [16]. Therefore, the circular and linear intermediates of retrozyme RNA detected in plants may be the result of both active RNA-RNA replication and long-distance transport in plants [15,16].

The above data suggest that during their existence in plant genomes, retrozyme circRNAs may have acquired specialized functions that remain to be identified and that activation of retrozyme transcription in response to stress may occur without changing promoter methylation levels, as shown in the case of the LTR-retrotransposon *ONSEN* under heat stress [16,17]. The activation of retroelement transcription in response to stress was demonstrated in a model of several other LTR-containing plant retrotransposons. In particular, the *AtCOPIA4* promoter located in the LTR has been shown to be activated in response to infection, probably due to the presence of binding sites for WRKY transcription factors, which are known to orchestrate transcriptional reprogramming during pathogen-associated molecular pattern-triggered immunity [18]. The 5′ LTRs of two *Phyllostachys edulis* LTR retrotransposons, PHRE1 and PHRE2, contain promoters that are activated during the response to heat stress by interaction with heat-dependent transcription factors [19]. However, in the case of retrozyme promoters, such regulation has yet to be elucidated.

In this study, we identified the transcription start site of the promoter contained in the LTR of *Nicotiana benthamiana* retrozyme 1 (NbRZ1) and mapped the promoter region essential for its transcriptional activity. The NbRZ1 transcript was further shown to potentially encode a protein that is targeted for proteasomal degradation in the plant cell. Using a viral expression vector, it was shown that overexpression of this protein in plants causes severe necrosis.

## 2. Results

### 2.1. Mapping of the NbRZ1 Transcription Start Site

**Figure 1:**
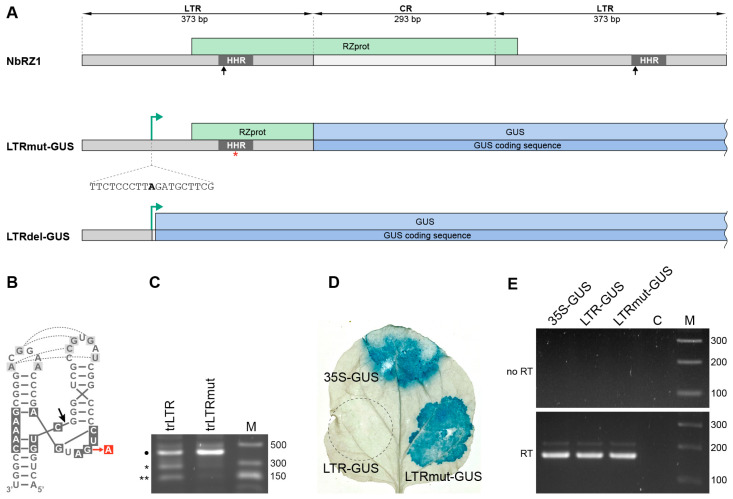
Identification of the transcription start site of the promoter contained in the LTR of NbRZ1. (**A**) Schematic representation of NbRZ1 and reporter constructs LTRmut-GUS and LTRdel-GUS. The positions and lengths of the LTRs and the central region (CR) are indicated. The hammerhead ribozyme (HHR) is shown in dark gray, and the black arrow points to the site of autocatalytic cleavage. The red asterisk indicates a point mutation in HHR that blocks autocatalytic cleavage. The position of the mapped transcription start site is indicated by the green arrow; the inset with the dotted line shows the fragment of the LTR sequence in which the mapped transcription start site at the LTR NbRZ1 promoter is shown in bold. The green box indicates the NbRZ1-encoded polypeptide RZprot. The GUS coding sequence and the GUS protein are shown in dark and light blue, respectively. (**B**) Structural model of the NbRZ1 hammerhead ribozyme. Residues forming the catalytic center are shown in dark gray boxes. Dotted lines indicate non-canonical interactions in the ribozyme structure. The red arrow indicates a point mutation that results in inhibition of NbRZ1 HHR self-cleavage. The site of autocatalytic cleavage is indicated by the black arrow. (**C**) In vitro analysis of ribozyme processing efficiency. The transcripts of LTRmut (trLTRmut) and LTRwt (trLTR) sequences were used for RNA mobility analysis in agarose gel: M, RNA size markers. The black dot indicates an unprocessed transcript, while the asterisks indicate autocatalytic cleavage products. (**D**) X-Gluc-stained leaf of *N. benthamiana* infiltrated with agrobacterial cultures carrying the LTR-GUS, LTRmut-GUS, and 35S-GUS constructs. (**E**) Total RNA from *N. benthamiana* leaves infiltrated with agrobacterial cultures carrying the LTR-GUS, LTRmut-GUS, and 35S-GUS constructs was analyzed by RT-PCR using GUS-specific primers. No RT, amplification carried out without reverse transcription; C, a control reaction without template; M, DNA size markers.

The NbRZ1 LTR region (Figure 1A) has been shown to contain a promoter; however, previous attempts to map the NbRZ1 transcription start site failed since, in plant cells, the LTR-directed transcript was efficiently processed by the hammerhead ribozyme (HHR) contained in it, and the 5′-proximal transcript region was cleaved off, preventing identification of the 5′-terminal transcript residue [15]. Therefore, to map the NbRZ1 transcription start site, a point mutation was introduced into the cloned NbRZ1 LTR region to inhibit ribozyme self-cleavage activity. The G to A substitution introduced into the NbRZ1 LTR ribozyme by site-directed mutagenesis (Figure 1B) has been previously shown to completely inhibit the self-cleavage ability of HHRs [20]. The resulting construct was termed LTRmut. The effect of the mutation on NbRZ1 ribozyme activity was analyzed in vitro using a T7 transcript of LTRmut. A transcript of the wild type (wt) LTR was used as a control. After the transcription reaction, RNA samples were analyzed in an agarose gel. Under in vitro transcription conditions, the wt transcript was found to be partially self-cleaved, giving rise to two transcript fragments of the expected size, whereas in the case of the LTRmut transcript, cleavage was significantly inhibited (Figure 1C).

To map the NbRZ1 transcription start site, the construct LTRmut-GUS was obtained, in which the GUS (*Escherichia coli* β-glucuronidase) coding region was cloned in a binary vector under the transcriptional control of LTRmut (Figure 1A). A similar LTR-GUS construct was obtained using the wt LTR region. Leaves of *N. benthamiana* plants were infiltrated with agrobacterial cultures carrying the LTR-GUS and LTRmut-GUS constructs. As an additional control, agroinfiltration was carried out with a culture carrying the 35S-GUS construct containing the strong constitutive 35S promoter of the *Cauliflower mosaic virus*. At 3 days post infiltration (dpi), the infiltrated leaves were stained for GUS activity with X-gluc, a GUS chromogenic substrate. After staining, a strong blue coloring was clearly visible in leaf areas agroinfiltrated for expression of LTRmut-GUS and 35S-GUS but not in the LTR-GUS-infiltrated area (Figure 1D). Reverse transcription (RT)-PCR analysis confirmed the presence of GUS transcripts in the areas infiltrated for the expression of 35S-GUS, LTR-GUS, and LTRmut-GUS (Figure 1E). These observations show that the LTR-GUS transcript is untranslatable, likely as a result of ribozyme self-cleavage, whereas the LTRmut-GUS transcript can be translated because it lacks the functional ribozyme. Thus, the mutation introduced in the NbRZ1 LTR to give LTRmut efficiently blocks ribozyme-dependent cleavage of the LTR-directed transcript in vivo. In addition, the efficient translation of the LTRmut-GUS-derived mRNA implies that it can have both the 5′-cap structure and the 3′-poly(A) tail, suggesting that the NbRZ1 LTR promoter-driven transcription is carried out by RNA polymerase II.

To determine the NbRZ1 transcription start site, 5′-RACE (rapid amplification of cDNA ends) was performed with GUS-specific primers on total RNA isolated at 3 dpi from *N. benthamiana* leaves agroinfiltrated for LTRmut-GUS expression, and the amplification product was cloned. Sequencing of five independent clones revealed identical sequences; in all clones, the 5′-end of the LTR-specific transcript was mapped to an adenosine residue corresponding to position 116 of the NbRZ1 LTR (Figure 1A).

### 2.2. Analysis of Retrozyme Expression in Plants

**Figure 2:**
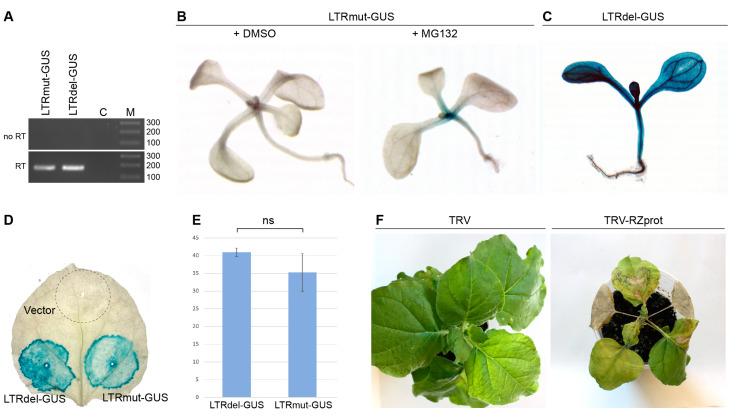
Expression of NbRZ1 in plants. (**A**) Total RNA from *A. thaliana* transgenic lines LTRmut-GUS and LTRdel-GUS analyzed by RT-PCR using GUS-specific primers. No RT; amplification carried out without reverse transcription; C, control reaction without template, M, DNA size markers. (**B**) MG132 proteasome inhibitor treatment and X-Gluc staining of *A. thaliana* LTRmut-GUS transgenic plants. Left, the plant treated with DMSO. Right, plant treated with MG132. (**C**) X-Gluc staining of *A. thaliana* LTRdel-GUS transgenic plant. (**D**) X-Gluc stained leaf of *N. benthamiana* infiltrated with agrobacterial cultures carrying the LTRmut-GUS, LTRdel-GUS, and empty vector constructs. (**E**) Quantitative RT-PCR analysis of GUS RNA levels in *N. benthamiana* leaves infiltrated with agrobacterial cultures carrying the LTR-GUS and LTRmut-GUS constructs. Bars show mean values and standard error; ns indicates no statistically significant difference between LTRmut-GUS and LTRdel-GUS (*p* > 0.05). (**F**) Analysis of the effects of the NbRZ1-encoded polypeptide RZprot on plants using a viral expression vector based on the genome of TRV. *N. benthamiana* plants infiltrated with cultures carrying the TRV and TRV-RZprot are shown.

Transgenic *Arabidopsis thaliana* plants were generated using the LTRmut-GUS construct to analyze retrozyme expression in plants further. In transformed plants, for which the presence of the LTRmut-GUS transgene was confirmed by PCR, GUS-specific mRNA was readily detected by RT-PCR with GUS-specific primers (Figure 2A). However, GUS enzymatic activity could not be detected by GUS staining in these plants (Figure 2B).

NbRZ1 contains an open reading frame (ORF) that potentially encodes a 19 kDa polypeptide designated as RZprot (Figure 1A). In the LTRmut-GUS construct, the GUS coding sequence is fused in-frame to a 5′-proximal portion of the RZprot ORF (Figure 1A). Therefore, translation of the LTRmut-GUS transcript could result in a translational fusion of the N-terminal region of RZprot and the GUS protein (Figure 1A). As the LTRmut-GUS transcript, but not a product of its translation, was detected in the transgenic plants, we hypothesized that the RZprot moiety of the RZprot-GUS fusion protein could be targeted for proteasome degradation resulting in degradation of the entire RZprot-GUS protein, as shown for other functional important proteins [21,22,23]. To verify this hypothesis, seedlings of LTRmut-GUS transgenic plants were treated with MG132, a potent cell-permeable proteasome inhibitor [24]. Further GUS staining of MG132-treated seedlings produced a blue coloring (Figure 2B), confirming, therefore, that the absence of LTRmut-GUS translation product in transgenic plants was caused by its proteasome degradation. As this product was readily detected upon agrobacteria-mediated transient LTRmut-GUS expression (Figure 1D), we suggest that its proteasomal degradation might be rather inefficient and, therefore, manifested only under the conditions of low-level LTRmut-GUS expression in transgenic plants. It should be noted that the GUS gene product is very stable in plants, and staining intensities can increase over time even when expression levels of the gene of interest are low [25]. This may explain why the LTRmut-GUS and LTRdel-GUS transgenic lines show different levels of staining. In the case of LTRmut-GUS plants, the corresponding translation product was degraded and did not accumulate efficiently in the plants. On the other hand, these data suggest that the RZprot ORF can be expressed in plants, providing a first indication of the retrozyme protein-coding capacity. Further detailed analyses using RZprot-specific antibodies are required to determine whether RZprot is indeed expressed in plants.

To confirm that the proteasomal degradation of the RZprot-GUS fusion protein was directed by the RZprot-specific sequence and to further characterize the NbZR1 LTR promoter, another construct was used to generate transgenic plants. In this construct, termed LTRdel-GUS, the GUS coding sequence was cloned under the control of the NbRZ1 LTR region located upstream of the mapped transcription start site (Figure 1A). In LTRdel-GUS, the RZprot coding region was completely deleted. GUS staining of seedlings of LTRdel-GUS transgenic plants resulted in the appearance of blue coloring (Figure 2C), indicating that the GUS protein lacking the RZprot fragment is not susceptible to proteasomal degradation. On the other hand, these observations show that the 115 bp region of the NbRZ1 LTR located upstream of the transcription start is sufficient for promoter activity. To compare the transcriptional efficiency of the full-length and truncated LTR, *N. benthamiana* leaves were agroinfiltrated for expression of LTRmut-GUS and LTRdel-GUS. GUS staining of infiltrated leaves at 3 dpi revealed similar levels of GUS expression (Figure 2D). Quantitative RT-PCR analysis of GUS RNA levels revealed no statistically significant difference between LTRmut-GUS and LTRdel-GUS (*p* > 0.05). (Figure 2E). These data demonstrate that the major elements of the NbRZ1 promoter are located upstream of the transcription start site.

To analyze the possible effects of the putative NbRZ1-encoded polypeptide RZprot on plants, the RZprot coding sequence was cloned into a viral expression vector based on the genome of *Tobacco rattle virus* (TRV) [26]. As the RZprot ORF contained the ribozyme (Figure 1A) that would mediate self-cleavage of the corresponding RNA in plants, a mutant version of the RZprot ORF was used that carried the point mutation described above for LTRmut; this mutation did not alter the encoded RZprot protein sequence. *N. benthamiana* plants were agroinfiltrated for expression of TRV-RZprot, and the TRV vector was used as a control. Like the TRV vector, TRV-RZprot caused systemic infection of plants; however, the infection symptoms induced by TRV-RZprot were drastically different from those induced by TRV. In control, TRV-infected plants, slight stunting, mild mosaic/yellowing of upper leaves, and occasional necrosis along the leaf veins were observed. In contrast to TRV, TRV-RZprot caused severe necrosis of agroinfiltrated and upper systemically infected leaves (Figure 2F). These observations suggest that high-level expression of RZprotis is detrimental to plants. Therefore, we hypothesize that the expression of RZprot if it occurs under native conditions, can only be at low levels or tightly regulated in a spatial/temporal manner, or both.

## 3. Discussion

Transposition of retroelements involves transcription of their genomic loci, translation of the resulting mRNA to produce proteins with the required enzymatic activities, reverse transcription leading to the synthesis of double-stranded DNA, and its integration into a new genomic locus [27]. Thus, the key event that initiates the act of transposition is the transcription of a retroelement genomic locus. In LTR-containing retrotransposons, as well as in retroviruses that also contain LTRs, transcription of their genomic loci is controlled by RNA polymerase II promoters located in LTRs [28,29]. In plants, transcriptional activation of such promoters can occur in response to stress, as has been demonstrated for a number of retroelements [17,18,19]. In this work, we have characterized the promoter contained in the LTR of the retrozyme NbRZ1, a non-autonomous retrotransposon of *N. benthamiana*. The transcription start site was mapped in the NbZR1 LTR; however, the mechanisms of regulation of the NbZR1 LTR promoter remain undiscovered. It should be noted that regulation of NbZR1 LTR promoter-driven transcription is expected based on the data showing that the NbZR1 LTR is highly methylated [15] likely prevents transcription of this locus, while a substantial amount of retrozyme RNA has been detected in *N. benthamiana* plants [15], indicating that transcription of NbRZ1 and other retrozyme genomic loci may occur under as yet unidentified conditions and is therefore likely to be subject to regulation. As demonstrated in the case of the LTR-retrotransposon *ONSEN*, activation of transcription of LTR-containing retrotransposons can occur without changing promoter methylation levels. Although the exact mechanism of such activation in the case of retrozymes and *ONSEN* has not yet been elucidated, it is conceivable that it could be associated with proteins containing a methyl CpG-binding domain (MBD), which bind chromatin regions with high levels of CG methylation preventing their activation, and that loss of MBD protein function leads to activation of transposable elements without altering the methylation level at the corresponding genomic loci [30,31]. On the other hand, since the circularized retrozyme RNA is capable of autonomous RNA-RNA replication [13,15], it remains to be established whether the retrozyme-specific RNA detected in plants is the result of transcription of genomic loci or autonomous replication. Further experiments are needed to determine whether the retrozyme LTR promoter-directed transcription is activated under specific conditions or at a certain developmental stage.

The retrozyme RNA contains an ORF that may encode a 19 kDa polypeptide called RZprot; expression of this protein has not been demonstrated, and its potential functions have remained unknown. The experiments reported here suggest that RZprot can be translated using the NbRZ1 RNA template. In addition, an N-terminal portion of RZprot fused to GUS was found to induce proteasomal degradation of the fusion protein, suggesting that the accumulation of RZprot in plant cells is tightly controlled. Retrozyme RNA exists in both linear and circular forms in plants [13,15], so it is unclear which of these can serve as a template for RZprot translation. In fact, circRNAs, although apparently lacking the 5′-cap and 3′-poly(A), can still serve as templates for translation that can be initiated in a cap-independent manner due to internal ribosome entry sites (IRES) or translation factors that bind to modified RNA residues [11,32,33,34]. The possibility of retrozyme circRNA translation is consistent with two available observations. First, the retrozyme circRNA is likely to be rather stable in plant cells since it has been shown, using *Peach latent mosaic viroid* as a model, that the HHR of the same type as in retrozymes most likely adopts an inactive conformation upon circularization, thus preventing its self-cleavage [35,36]. Second, the HHR tertiary structure can serve as an IRES-like element and is distantly topologically related to the structure of the picornavirus IRES [37]. Therefore, the putative translation of retrozyme RNA can be considered as rather possible and requires further experimental evaluation. On the other hand, the translation of RZprot on the template of the linear retrozyme transcript seems less probable since such a transcript can only be translated if it avoids HHR self-cleavage, which is known to be as efficient as 94% at magnesium ion concentrations similar to those in living cells, as has been found for a viroid HHR similar in structure to the retrozyme HHR [38]. It should also be kept in mind that only a product of retrozyme genomic locus transcription can be translated as a linear RNA in a 5′-cap/3′-polyA-dependent manner, as the products of autonomous retrozyme RNA-RNA replication, both linear and circular, apparently lack these terminal structures. Further research is needed to determine whether circular and linear retrozyme forms can be translated in plants.

The data presented in this paper and previously published data on retrozyme biology are consistent with the following model. The activity of the retrozyme LTR promoter and the transcription of retrozyme genomic loci are generally inhibited by DNA methylation and occur only at specific developmental stage(s) or in response to specific stimuli; the synthesized retrozyme RNA can persist in plants due to RNA-RNA replication as well as systemic transport and have as yet unidentified functions [16]. One of these functions may be related to the findings presented here, indicating that the retrozyme RNA has a coding capacity and may be translated to a protein of unknown function. The observations that overexpression of this protein leads to extensive necrosis of plant tissues and that this protein is targeted for proteasomal degradation demonstrate tight regulation of its expression that may indicate its functional importance.

In view of the data presented in this paper, future studies of retrozymes in plants should aim at a more detailed analysis of their transcription and translation.

## 4. Materials and Methods

### 4.1. Plant Material

*N. benthamiana* plants were grown from seed under standard conditions (16 h/8 h light/dark cycles, 24/20 °C day/night temperatures, 50% humidity) in a glasshouse. The 3 to 5-week-old plants were used for agrobacterial-mediated transient protein expression. *A. thaliana* plants were grown from seed after vernalization at 4 °C for 3 days in growth chambers under standard conditions (22/20 °C day/night temperatures, 60–70% relative humidity, and 8 h/16 h light/dark cycles).

### 4.2. Plant Agroinfiltration

*Agrobacterium tumefaciens* (strain C58C1 or GV3101) cells were transformed with binary vectors using a freeze–thaw method. Overnight cultures of agrobacteria were grown in the Luria–Bertani (LB) medium at 28 °C with selective antibiotics, 10 mM 2-(*N*-morpholino)ethanesulfonic acid (MES) monohydrate (Sigma-Aldrich, Saint Louis, MO, USA), pH 5.5, and 20 μm acetosyringone (Sigma-Aldrich, Saint Louis, MO, USA). 500 µL of overnight culture was transferred to 5 mL of fresh LB medium at 28 °C with selective antibiotics 10 mM MES, pH 5.5, and 20 μm acetosyringone for 5 h. Cells were pelleted by centrifugation, resuspended in the infiltration medium (10 mM MES, pH 5.5, 10 mM MgCl_2_ (Sisco Research Laboratories Pvt. Ltd., New Mumbai, India), and 150 mM acetosyringone) and incubated at room temperature for 3–4 h. Prior to infiltration, *A. tumefaciens* suspensions were diluted to a final OD_600_ = 0.3. The agrobacterial cultures were infiltrated onto the abaxial surface of *N. benthamiana* leaves using a needleless syringe.

### 4.3. Molecular Cloning and Recombinant Constructs

To obtain the pLH-LTRwt:GUS construct, the NbRZ LTR sequence was amplified on the template of the previously published construct NbRZ1 [15] with specific primers (Supplementary Table S1). The resulting amplification product was digested with *Eco*RI-*Nco*I (ThermoFisher Scientific, Waltham, MA, USA) restriction endonucleases and cloned into the pLH* binary vector [39] carrying the GUS coding sequence. For the pLH-LTRmut:GUS construct, the NbRZ LTR sequence was amplified by overlap PCR with appropriate primers (Supplementary Table S1) on the pLH-LTRwt:GUS construct and cloned with *Eco*RI-*Nco*I restriction endonucleases into the pLH* binary vector carrying the GUS coding sequence. The resulting constructs were then used by agroinfiltration for transient expression in N. benthamiana plants.

To generate transgenic *A. thaliana* plants, the pCambia-LTRmut:GUS construct was obtained. For this, the expression cassette from pLH-LTRmut:GUS was subcloned into pCambia1300 binary vector using *EcoR*I-*Xba*I (ThermoFisher Scientific, Waltham, MA, USA) restriction endonucleases. To obtain the pCambia-LTRdel:GUS construct, the LTR region located upstream of the mapped transcription start site was amplified with specific primers (Supplementary Table S1) on the pLH-LTRmut:GUS template. The resulting amplification product was digested with *EcoR*I-*Nco*I restriction endonucleases and cloned into the pCambia-LTRmut:GUS.

To obtain TRV-RZprot using overlap PCR. In the first step, one PCR product was obtained, using previously described NbRZ1 [15] as a template and RZ-Eco-Nco-P and NbRZ1-LTR-mut-ovl-M (Supplementary Table S1) as primers, and another PCR product was obtained from NbRZ1 using primers TRV-RZ-M and NbRZ1-LTR-mut-ovl-P (Supplementary Table S1). In the second step, two PCR products were fused by amplification with primers RZ-Eco-Nco-P and TRV-RZ-M. The resulting DNA fragment was digested with *EcoR*I-*Sal*I (ThermoFisher Scientific, Waltham, MA, USA) and cloned in a TRV RNA2-based vector [40] digested with *Eco*RI-*Xho*I (ThermoFisher Scientific, Waltham, MA, USA). The recombinant construct for the transient expression of TRV1 was described previously [41]. All obtained constructs were verified with sequencing.

### 4.4. 5′-RACE

The 5′-RACE analysis was carried out using the Mint RACE kit (Evrogen, Moscow, Russia). This protocol employs the Step-Out RACE procedure, as described in detail earlier [42]. The reverse transcription and subsequent amplification steps were performed according to the manufacturer’s instructions, using the primers included in the kit and gene-specific primers (Supplementary Table S1). PCR products were then cloned into the pAL2-T vector (Evrogen, Moscow, Russia) and sequenced.

### 4.5. GUS Staining

Infiltrated *N. benthamiana* leaves and 2.5-week-old *A. thaliana* seedlings were vacuum-infiltrated and stained overnight at 37 °C in GUS staining buffer (containing 0.05 M sodium phosphate buffer (pH 7.0), 0.2% Triton X-100 (Suzhou Yacoo Science Co., Ltd., Suzhou, Jiangsu, China), 0.5 mM potassium ferrocyanide (LenReactiv, Saint-Petersburg, Russia), 0.5 mM potassium ferricyanide (LenReactiv, Saint-Petersburg, Russia) and 1 mM X-Gluc (Chem-Impex, Wood Dale, IL, USA)). The samples were then incubated for up to 8 h in 95% ethanol and observed.

### 4.6. Transgenic Lines Generation

The generation of *A. thaliana* transgenic lines was performed using the *A. tumefaciens* strain GV3101 containing the vector pCambia-LTRmut:GUS or pCambia-LTRdel:GUS; the transformation of *A. thaliana* (Col-0) plants was carried out by the floral-dip method [43]. Transformants were screened on 1/2 MS media containing 50 μg/mL hygromycin B (Sisco Research Laboratories Pvt. Ltd., New Delhi, India) and then transferred to soil. Transgenic lines selected from T3 lines were subjected to histochemical staining.

### 4.7. 26S Proteasome Inhibition

The inhibition of the 26S proteasome in *A. thaliana* seedlings was performed in an MS liquid medium supplemented with 150 µM MG132 (Sigma-Aldrich, Saint Louis, MO, USA) for 12 h. MG132 stock solution (5 mg/mL) was prepared with dimethylsulfoxide (DMSO) (Sigma-Aldrich, Saint Louis, MO, USA). The seedlings were incubated in an MS liquid medium supplemented with DMSO as a control.

### 4.8. In Vitro Transcription and Ribozyme Processing

RNA used for in vitro ribozyme processing experiments was obtained by in vitro transcription with T7 RNA polymerase (ThermoFisher Scientific, Waltham, MA, USA). The transcripts containing LTRmut and LTRwt sequences were produced by transcription on templates of PCR products containing T7 promoter, which were obtained using the appropriate primers (Supplementary Table S1) using pLH-LTRmut:GUS or pLH-LTRwt:GUS as templates. For analysis of ribozyme processing efficiency, in vitro transcription products were incubated in formamide loading buffer (deionized formamide (Sigma-Aldrich, Saint Louis, MO, USA) 80% (*w*/*v*), EDTA (Sisco Research Laboratories Pvt. Ltd., New Mumbai, India) (pH 8.0) 10 mM, bromophenol blue (LenReactiv, Saint-Petersburg, Russia) 1 mg/mL) at 70 °C for 15 min for RNA denaturation. The samples were then chilled on ice and analyzed in 2% agarose gel.

### 4.9. Total RNA Extraction, Reverse Transcription and Quantitative PCR

Leaf disks were frozen in liquid nitrogen and ground to fine powder. Total RNA was extracted using ExtractRNA reagent (Evrogen, Moscow, Russia) according to the manufacturer’s instructions. To prevent contamination with plant genomic DNA, samples were thoroughly treated with RNase-free DNAseI (ThermoFisher Scientific, Waltham, MA, USA). The total RNA samples (1 µg each) were transcribed into cDNA with oligo(dT) primer using a Revertaid reverse transcriptase (ThermoFisher Scientific, Waltham, MA, USA). Then, 1 μL of three-fold-diluted reverse-transcription product was used for real-time PCR reactions with a qPCRMix M-440 (Synthol, Moscow, Russia) using GUS-specific primers or primers specific for F-box mRNA, which was used as the reference gene (Supplementary Table S1). Reactions were carried out in the CFX Connect Real-Time PCR System (Bio-Rad, Hercules, CA, USA) The *C*_t_ value for GUS RNA was normalized to the reference gene mRNA. For the statistical analyses of data, a nonpaired two-tailed Student’s *t*-test was applied.

## Data Availability

Data are contained within the article and Supplementary Materials.

## Supplementary Materials

The following supporting information can be downloaded at: https://www.mdpi.com/article/10.3390/plants14081205/s1, Table S1: Primers used in this study.
