# Supplementary material for: Expression of the Nicotiana benthamiana Retrozyme 1 (NbRZ1) Genomic Locus
Source: Plants (Basel). 2025 Apr 14;14(8):1205. doi: 10.3390/plants14081205 (PMC12029980; doi:10.3390/plants14081205)
Supplement: Supplementary file 1 [file plants-14-01205-s001.zip › plants-3531143-supplementary.pdf]

Table S1. Primers used in this study

**LTR-GUS cloning**	
NbRZ1-LTR-P	CGCGAATTCTGTCACGACCCGATTGTCGTG
NbRZ1-LTR-mut-ovl-P	GCTCCCCTAATGAGCCCAAG
NbRZ1-LTR-mut-ovl-M	CTTGGGCTCATTAGGGGAGC
NbRZ1-LTR-M	CCGCCATGGTGTCACGGGACGAGAGTCCG
NbRZ1-LTRdel-M	GCGCCATGGCACCGCCGCGCCTTGCGGAGTAAGGGAGAACGCCTTATCGC
**5´RACE**	
NbRZ1-GUS-RACE-out	GAATGCCCACAGGCCGTC
NbRZ1-GUS-RACE-in	CGGACCATGGTGTCACGG
**GUS RNA detection**	
GUSdet-P2	CAACTCCTACCGTACCTCGCATTA
GUSdet-M2	GCCTCTTCGCTGTACAGTTCTTTC
**In vitro transcription**	
T7-NbRZ1-P	CGTAATACGACTCACTATAGGGACGTGTCTACATTCACGTCCA
NbRZ1-LTR-M	CCGCCATGGTGTCACGGGACGAGAGTCCG
**TRV cloning**	
RZ-Eco-Nco-P	CCGGAATTCACCATGGACTTGACAATGAAGGG
TRV-RZ-M	CCCGTCGACTCATTTGGTCTTTACGGTCACG
NbRZ1-LTR-mut-ovl-P	GCTCCCCTAATGAGCCCAAG
NbRZ1-LTR-mut-ovl-M	CTTGGGCTCATTAGGGGAGC
**qRCR**	
GUSdet-P1	GTGAAGAGTATCAGTGTGCATGGC
GUSdet-M1	TTTTCACCGAAGTTCATGCCAGTC
F-Box-F	GGCACTCACAAACGTCTATTTC
F-Box-R	ACCTGGGAGGCATCCTGCTTAT
